# The accuracy of a newly developed guide system in medial meniscus posterior root repair: a comparison between two aiming guides

**DOI:** 10.1186/s43019-019-0007-1

**Published:** 2019-08-07

**Authors:** Takayuki Furumatsu, Yuki Okazaki, Yuya Kodama, Yoshiki Okazaki, Yusuke Kamatsuki, Shin Masuda, Takaaki Hiranaka, Toshifumi Ozaki

**Affiliations:** Department of Orthopaedic Surgery, Okayama University Hospital, 2-5-1 Shikata-cho, Kita-ku, Okayama, 700-8558 Japan

**Keywords:** Knee, Medial meniscus, Root tear, Pullout repair, Tibial guide

## Abstract

**Purpose:**

Posterior root repair of the medial meniscus (MM) can prevent rapid progression of knee osteoarthritis in patients with a MM posterior root tear (MMPRT). The anatomic reattachment of the MM posterior root is considered to be critical in a transtibial pullout repair. However, tibial tunnel creation at the anatomic attachment is technically difficult. We hypothesized that a newly developed point-contact aiming guide [Unicorn Meniscal Root (UMR) guide] can create the tibial tunnel at a better position rather than a previously designed MMPRT guide. The aim of this study was to compare the position of the created tibial tunnel between the two meniscal root repair guides.

**Materials and methods:**

Thirty-eight patients underwent transtibial pullout repairs. Tibial tunnel creation was performed using the UMR guide (19 cases) or MMPRT guide (19 cases). Three-dimensional computed tomography images of the tibial surface were evaluated using the Tsukada’s measurement method postoperatively. The expected anatomic center of the MM posterior root attachment was defined as the center of three tangential lines referring to three anatomic bony landmarks (anterior border of the posterior cruciate ligament, lateral margin of the medial tibial plateau, and retro-eminence ridge). The expected anatomic center and tibial tunnel center were evaluated using the percentage-based posterolateral location on the tibial surface. The distance between the anatomic center and tunnel center was calculated.

**Results:**

The anatomic center of the MM posterior root footprint was located at a position of 79.2% posterior and 39.5% lateral. The mean of the tunnel center in the UMR guide was similar to that in the MMPRT guide (posterior direction, *P* = 0.096; lateral direction, *P* = 0.280). The mean distances between the tunnel center and the anatomic center were 4.06 and 3.99 mm in the UMR and MMPRT guide group, respectively (*P* = 0.455).

**Conclusions:**

The UMR guide, as well as the MMPRT guide, is a useful device to create favorable tibial tunnels at the MM posterior root attachment for pullout repairs in patients with MMPRTs.

**Level of evidence:**

IV

## Introduction

A medial meniscus (MM) acts as a secondary stabilizer against the anterior tibial shift and external rotation of the tibia [1, 2]. MM posterior root attachment has an important role in regulating the meniscal movement and hoop tension during knee motion and load-bearing. MM posterior root tears (MMPRTs) involved in complete radial and/or oblique tears adjacent to the root attachment lead to accelerated degradation of the knee joint cartilage by disrupting meniscal functions [3]. MM posterior root repair can reduce an excessive tibiofemoral contact pressure following the MMPRT by anchoring the MM posterior root and horn [4]. Several arthroscopic repair techniques, such as the transtibial pullout repair and suture anchor-dependent repair, show more favorable clinical outcomes compared with conservative treatments in patients with MMPRTs [5, 6].

In arthroscopic MM posterior root repairs, an accurate positioning of the tibial tunnel aperture seems to be critical in restoring meniscal function following transtibial pullout repair [5]. In a biomechanical study, 3-mm displacement of the meniscal attachment induces cartilage deformation by decreasing the meniscal hoop tension in a porcine meniscus root tear model [6]. A non-anatomic repair of the MM posterior root attachment cannot restore the tibiofemoral contact pressure in human cadaveric knees [4]. Therefore, the anatomic placement of the MM posterior root/horn is considered to be necessary for obtaining good clinical outcomes in patients with MMPRT following MM posterior root repair [7]. The attachment of the MM posterior root is located on a triangular area surrounded by the lateral border of the medial tibial plateau, posterior cruciate ligament (PCL), and retro-eminence ridge [8, 9]. Several studies report that the MM posterior root has its attachment at 9.6 mm posterior and 0.7 mm lateral to the apex of the medial tibial eminence [4, 7, 8]. In a three-dimensional (3D) computed tomography (CT) image analysis, an anatomic center of the MM posterior root attachment is located at a position of 78.5% posterior and 39.4% lateral [10] using Tsukada’s method [11]. However, tibial tunnel creation at the anatomic center of the MM posterior root attachment is technically difficult because of the narrow medial joint space and lack of absolute standard landmarks. A specially designed MMPRT aiming guide for transtibial pullout repair (Smith & Nephew, Andover, MA, USA) has an advantage in creating the tibial tunnel aperture at a more anatomic location compared with a conventional non-anatomically designed multi-use guide (Arthrex, Naples, FL, USA) [10]. The MMPRT guide has a narrow twisting/curving shape adjusted to the medial intercondylar space. However, the MMPRT guide does not have a tip-aiming hook to set a guide wire at an accurate point. In this study, we made a point-contact aiming guide [Unicorn Meniscal Root (UMR) guide, Arthrex] to achieve rigid positioning of the tibial tunnel center for the MM posterior root repair. We hypothesized that a newly developed point-contact UMR guide can create the tibial tunnel at a better position rather than a previously designed MMPRT guide. The aim of this study was to compare the tibial tunnel position between two meniscal root repair guides.

## Materials and methods

This study received the approval of our Institutional Review Board, and written informed consent was obtained from all patients. Thirty-eight patients (23 women and 15 men, a mean age of 63.2 years), who underwent transtibial pullout repairs for MMPRT between May 2018 and January 2019, were included (Table 1). All the patients had an episode of a sudden posteromedial painful popping, continuous knee pain, and complete radial/oblique MMPRT (meniscal root tear classification, types 2/4) [7, 12]. Patients who had radiographic knee osteoarthritis involved in Kellgren-Lawrence grade III or more and a previous history of meniscus injury or knee surgery were excluded. All the patients were diagnosed as having MMPRTs with magnetic resonance imaging (MRI) examinations and met operative indications for arthroscopic transtibial pullout repair (a femorotibial angle < 180°, Outerbridge grade I or II, and Kellgren-Lawrence grades 0–II) [13–18]. Duration from painful popping event to surgery was 84.4 ± 68.2 days. The presence of the MMPRT was defined according to characteristic MRI findings such as cleft, giraffe neck, ghost, radial tear, and meniscal extrusion signs of the MM posterior root within 9 mm from the attachment [19–21]. We divided the patients into two groups to compare the tibial tunnel position between the UMR guide (Arthrex) and the MMPRT guide (Smith & Nephew). We allocated 19 patients to each group according to the time period. In a power analysis (α error = 0.05, 1 − β error = 0.80), the required sample size was 16 patients in each group (difference, 2 mm; standard deviation, 2 mm). The types of the MMPRT were determined by careful arthroscopic examinations according to the meniscal root tear classification [22].

**Table 1:** Demographics and clinical characteristics

	UMR guide	MMPRT guide	*P* value
Number of patients	19	19	
Gender, men/women	7/12	8/11	
Root tear classification			
Types 1/2/3/4/5	0/16/0/3/0	0/17/0/2/0	
Kellgren-Lawrence grade			
Grades 0/I/II/III/IV	0/4/15/0/0	0/7/12/0/0	
Age, years	61.8 ± 8.7	64.5 ± 9.1	0.184
Height, m	1.58 ± 0.10	1.61 ± 0.11	0.225
Body weight, kg	67.7 ± 17.4	68.8 ± 17.7	0.420
Body mass index, kg/m^2^	26.6 ± 4.2	26.1 ± 3.7	0.355
Femorotibial angle, °	177.6 ± 1.5	177.0 ± 1.1	0.083
Duration from injury to surgery, days	79.0 ± 70.4	90.1 ± 67.6	0.323

Data of age, height, body weight, body mass index, and femorotibial angle are displayed as a mean ± standard deviation. *UMR* Unicorn Meniscal Root, *MMPRT* medial meniscus posterior root tear

### Surgical procedure

**Fig. 1:**
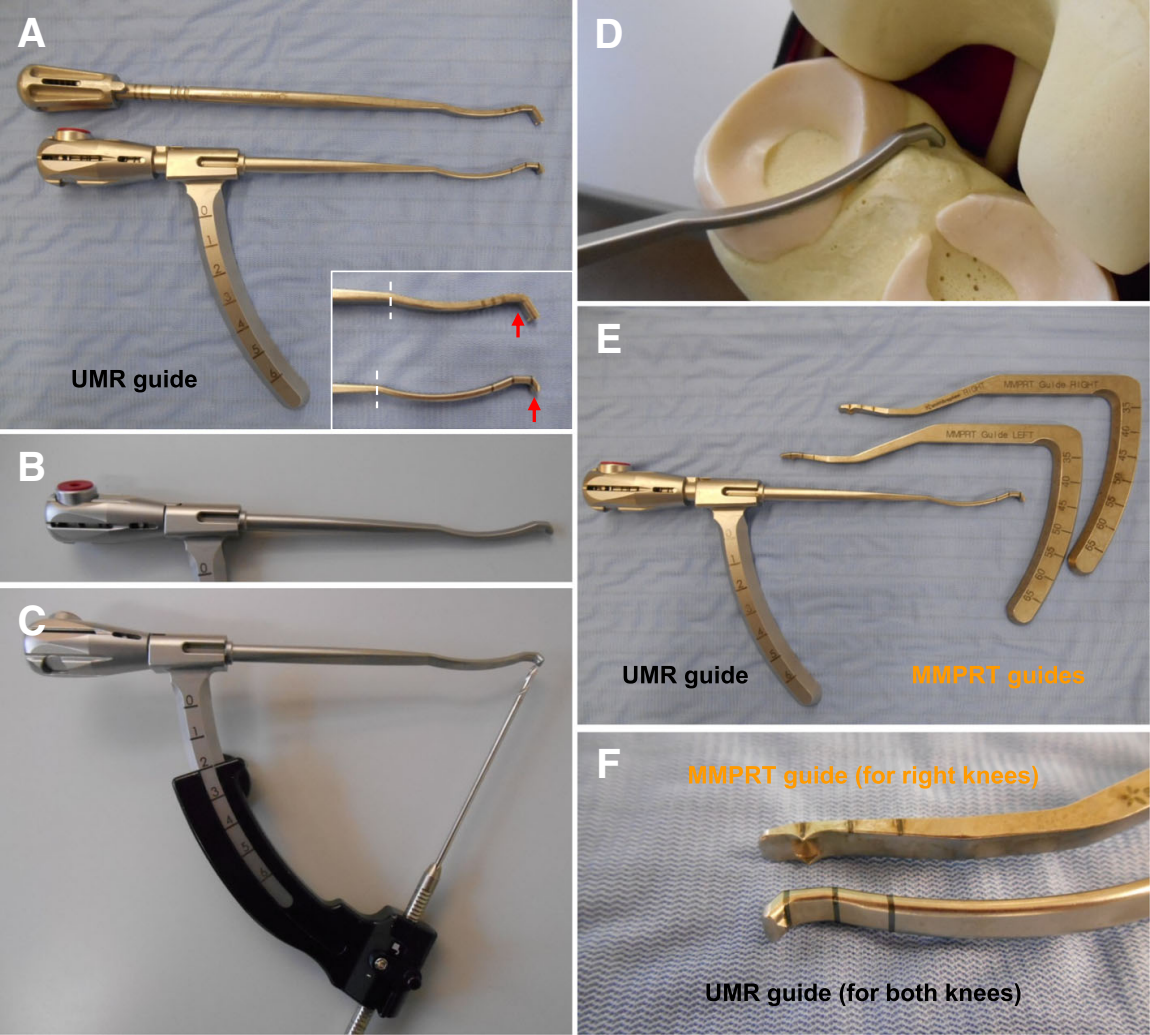
Aiming guides for medial meniscus (MM) posterior root repair. **a** A conventional meniscal root marking hook (upper, Arthrex). The Unicorn Meniscal Root (UMR) guide (lower). An inlet denotes the difference between each guide in shape, sizing scale, and aiming system. A guide-wire catching point (red arrows) is set at the tip of the UMR guide. The catching point of the conventional guide is set at the neck of the hook. The length of a curving part between a border (dashed lines) and catching point is longer in the UMR guide than in the conventional guide. The UMR guide has a more anatomic design to aim the native MM posterior root attachment compared with the conventional guide. **b** A push-button locking system for both sides of the knee. **c** A point-contact aiming system of the UMR guide. **d** The UMR guide has a narrow/slim curving shape based on an anatomic design. **e** The UMR and meniscus posterior root tear (MMPRT) guides. **f** The difference in a guide-wire catching system between the two guides

Standard anteromedial and anterolateral portals were used for the MM posterior root repairs. An outside-in pie-crusting technique involving a release of the deep medial collateral ligament was usually performed by using a standard 18-gauge needle [18]. The torn end of the MM posterior root/horn was grasped and repaired using the two-simple-stitches configuration [18, 23]. A knee Scorpion^TM^ suture passer (Arthrex) was used to pass No. 2 FiberWire (or FiberStick, Arthrex) sutures vertically through the MM posterior horn. Two FiberWire sutures were retrieved through the anteromedial portal. Tibial tunnel creation was performed using the UMR guide or the MMPRT guide [10]. The UMR guide was a newly developed aiming device that had a narrow/slim curving shape adjusted to the medial intercondylar space and included a push-button locking system for both sides of the knee (Fig. 1a-f). The aiming guides were placed at the MM posterior root attachment from the anteromedial portal with reference to the medial tibial eminence and PCL. A 2.4-mm guide wire was inserted, using the aiming device, at a 50° angle to the articular surface, and a 4.0-mm cannulated reamer was used to create a tibial tunnel. Two sutures were pulled out through the tibial tunnel. Tibial fixation was performed with the knee flexed at 20° and with an initial tension of 30 N using a 5.0 × 20-mm interference screw: ThreadTight (Arthrex) or Biosure RG (Smith & Nephew). The combination of the 4.0-mm tibial tunnel and 5.0-mm interference screw would not break the No. 2 FiberWire in patients with MMPRTs. The bone quality of the proximal tibia would be poor in the middle-aged patients who have MMPRTs. Thus, we used an interference screw instead of a Double Spike Plate (Meira, Aichi, Japan) for tibial fixation. An additional anchor screw (5.0 × 25-mm GTS cancellous screw, Meira) was inserted below the tibial tunnel aperture for stabilizing the sutures safely. All the surgical procedures were performed by a single experienced surgeon.

### 3D CT-based measurements

**Fig. 2:**
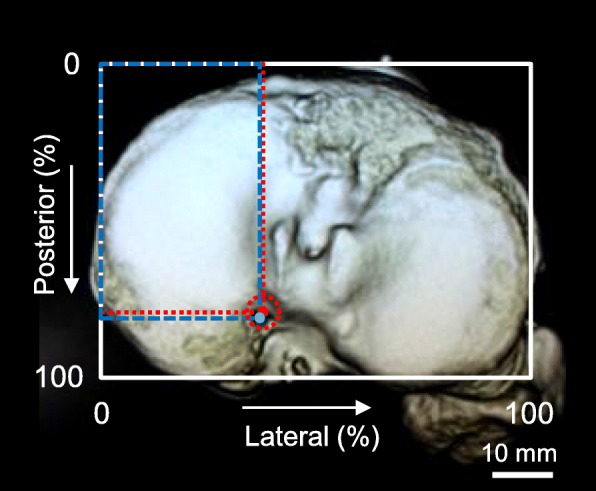
Distance between tibial tunnel center and expected anatomic center. The location on the three-dimensional computed tomography (3D CT)-based tibial surface was expressed as a posterolateral percentage using Tsukada’s method [11]. The anatomic center of the medial meniscus (MM) posterior root attachment was defined by the center of a circle (red dotted circle) that contacted three margins [anterior border of the posterior cruciate ligament (PCL) tibial attachment, lateral margin of the medial tibial plateau, and retro-eminence ridge]. Red dot, expected anatomic center of the MM posterior root attachment (in this example: 77.8% posterior and 38.5% lateral). Blue dot, tibial tunnel center (79.3% posterior and 37.1% lateral). The distance between the tibial tunnel center and anatomic center was 1.58 mm in this patient who underwent MM posterior root repair using the Unicorn Meniscal Root (UMR) guide

All patients underwent CT examination at 1 week postoperatively. CT images were obtained with an Asteion 4 Multislice CT System (Toshiba Medical Systems, Tochigi, Japan) using 120 kVp and 150 mA, and 1-mm slice thickness. CT reconstruction of the tibial condyles in the axial plane [24] was completed using a 3D volume-rendering technique (AZE Virtual Place software, Tokyo, Japan). 3D CT images of the tibial surface were evaluated using a rectangular measurement grid as described [11]. The image was rotated to visualize the superior aspect of the proximal tibia, with the internal/external rotation adjusted until the most posterior articular margins of both the medial and lateral tibial plateaus were placed on the horizontal level (Fig. 2). The location of interested points on the tibial surface was assessed using a percentage-dependent method. The posterolateral location on the tibial surface was expressed as a percentage using Tsukada’s method [11]. The expected anatomic center of the MM posterior root attachment was defined as a center of three tangential lines referring to three anatomic bony landmarks (anterior border of the PCL tibial attachment, lateral margin of the medial tibial plateau, and retro-eminence ridge) of the triangular footprint of the MM posterior root (Fig. 2). On 3D CT images, a virtual perfect circle that contacted these three tangential lines with the minimum radius was used to determine an expected anatomic center. The tangential line referring to each bony landmark was set at the nearest point to each expected anatomic center. Tibial tunnel centers were determined as the central point of the circular or oval tunnel aperture. The distance between the tunnel center and anatomic center was measured on 3D CT images (Table 2).

**Table 2:** Location of tibial tunnel center

	UMR guide	MMPRT guide	*P* value
Tibial tunnel center			
Posterior, %	74.5 ± 5.4	72.4 ± 4.0	0.096
Lateral, %	37.6 ± 2.9	38.1 ± 2.6	0.280
Distance between tunnel center and anatomic position, mm	4.06 ± 1.61	3.99 ± 1.99	0.455

Data are displayed as a mean ± standard deviation. *UMR* Unicorn Meniscal Root, *MMPRT* medial meniscus posterior root tear

### Statistical analysis

Data were presented as means ± standard deviations. Differences between groups were compared using the Mann-Whitney *U* test. Significance was set at *P* < 0.05. Two orthopaedic surgeons independently measured the location of expected anatomic center and tibial tunnel center. Each observer performed each measurement twice, at least 2 weeks apart. The inter-observer and intra-observer reliabilities were assessed with the intra-class correlation coefficient (ICC). An ICC > 0.80 was considered to represent a reliable measurement.

## Results

**Fig. 3:**
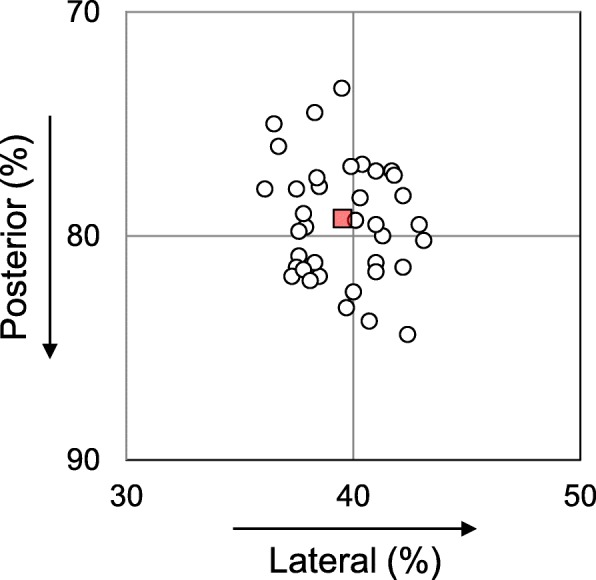
Location of expected anatomic center of the medial meniscus (MM) posterior root attachment (open dots). The mean of the MM posterior root anatomic center was at a position of 78.8% posterior and 39.4% lateral (red square) on a three-dimensional computed tomography (3D CT) image of the tibial surface

No significant differences between the UMR and MMPRT guide groups were observed in preoperative age, height, body weight, body mass index, and femorotibial angle (Table 1). The mean anatomic center of the MM posterior root attachment was located at a position of 79.2% posterior and 39.5% lateral (Fig. 3). The MM posterior root anatomic center was similar in each group (UMR guide, 79.8% posterior and 39.6% lateral position; MMPRT guide, 78.6% posterior and 39.4% lateral position). The values of the inter-observer and intra-observer reliabilities were considered high, with mean ICC values of > 0.91 and > 0.93, respectively.

**Fig. 4:**
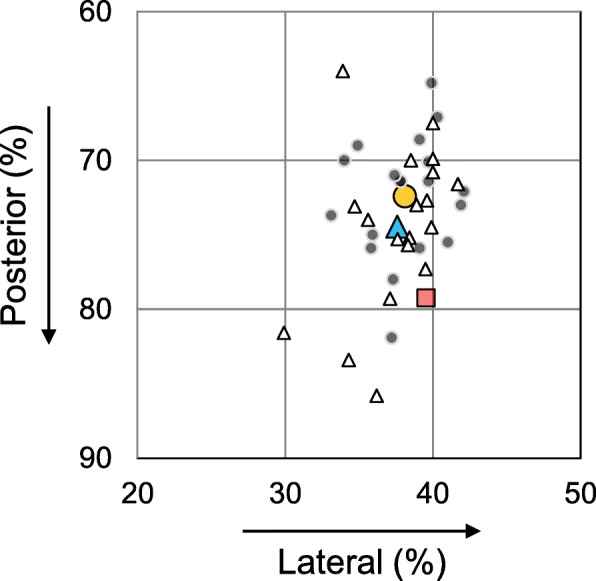
Locations of tibial tunnel centers and anatomic center. Red square: the mean anatomic center. Blue triangle: the mean of the tibial tunnel center created by the Unicorn Meniscal Root (UMR) guide (white triangles indicate the location of each case). Orange circle: the mean of the tibial tunnel center in the medial meniscus posterior root tear (MMPRT) guide group (gray dots indicate the location of each case)

The tibial tunnel center of the UMR guide group was located at a position of 74.5% ± 5.4% posterior and 37.6% ± 2.9% lateral (Table 2 and Fig. 4). In the MMPRT guide group, the tibial tunnel center was located at a position of 72.4% ± 4.0% posterior and 38.1% ± 2.6% lateral. Post-hoc power values between the two guide groups were 27.5% and 8.1% in the posterior and lateral directions of tunnel center positions, respectively. The distance between the tunnel center and anatomic center was 4.06 ± 1.61 mm and 3.99 ± 1.99 mm in the UMR and MMPRT guide groups, respectively (a post-hoc power, 3.3%; Table 2). No significant differences in tunnel center position and distance between the

two centers were detected between the UMR and MMPRT guide groups (Table 2).

## Discussion

This study demonstrated that tibial tunnel centers created using the point-contact UMR guide were similar to those in the MMPRT guide group. Our hypothesis that the UMR guide can create the tibial tunnel at a better position than the MMPRT guide was refuted. The tibial tunnel aperture was located at a favorable position in both groups during arthroscopic MM posterior root repairs. We propose that the newly developed UMR guide system has a high accuracy in creating tibial tunnels at reliable positions during the MM posterior root pullout repairs and does this as well as the MMPRT guide.

In our 3D CT-based measurements, the mean position of the tibial tunnel center in the UMR guide group was similar to that in the MMPRT guide group (Table 2 and Fig. 4). A power analysis did not show a statistical power to achieve the 0.05 level of significance in the tunnel center position towards the posterior direction (a post-hoc power, 27.5%). A more posterior setting of the tibial tunnel may induce a technical difficulty of suture relay. Remaining soft tissues around the posterior root attachment would obstruct an arthroscopic view for suture retrieval. Surgical techniques, such as the outside-in pie-crusting technique or medial collateral ligament release, will be required to obtain the medial joint space widening for accurate tunnel positioning and suture passage during the MM posterior root repair.

MMPRTs lead to abnormal biomechanics of the tibiofemoral joint and the inability to convert axial loads into hoop stresses [25, 26]. Repair of the MMPRT has been shown to reduce the mean tibiofemoral contact pressure by increasing the tibiofemoral contact area in a human cadaveric knee study [27]. Several authors have reported that an anatomic repair of the MM posterior root may be critical for restoring the biomechanical function of the MM [4, 6, 7]. However, there has been no clinical evaluation involved in the relationship between tibial tunnel location and postoperative outcomes following the MM posterior root repair. In addition, patients have their own tibial plateau sizes and their specific MM posterior root attachment. In our study, the distance between the tibial tunnel center and expected anatomic center of the MM posterior root attachment was approximately 4 mm in transtibial pullout repairs using the specially designed UMR and MMPRT guides (Table 2). Previous studies demonstrate that an average attachment area of the MM posterior root is 30.4–47.3 mm^2^ and the MM posterior root attachment forms an oval or triangular shape [8, 28–30]. We consider that the distance of 4-mm between tunnel center and anatomic center would be acceptable because the radius of the provisional circle to determine the expected anatomic center was 4–5 mm on 3D CT images (Fig. 2).

The MMPRT guide has several advantages in creating a favorable tibial tunnel during pullout repairs in patients with MMPRTs [10]. The narrow and anatomically curving shape of the MMPRT guide can help us to set a guide wire at a more accurate position with high reproducibility compared with previously designed meniscal root guides. The UMR guide has a more anatomic design and longer curving arm to insert the guide into a narrow joint space if the patient has a long anteroposterior distance between the anteromedial portal and the MM posterior root attachment (Fig. 1d-f). In addition, the UMR guide can enable us to set a guide wire more posteriorly because of its point-contact aiming system. On the other hand, the MMPRT guide has a wider safety margin to protect guide wire penetration at the tip of the guide (Fig. 1f). Although the MMPRT guide is separately provided for the left and right knees, the surgeon-friendly UMR guide has an all-in-one and free-aiming system for the medial joint space of both knees. We believe that the UMR guide may have some superiority to the MMPRT guide in tibial bone tunnel creation during the MM posterior toot repair.

There are several limitations to this study. First, the sample size was small. A further study with a larger sample size will be required. Second, the relationship between the tibial tunnel position and postoperative clinical outcome was not evaluated. Third, there was a possibility that an ideal point of the tibial tunnel might be different from the expected anatomic center on 3D CT images. In addition, the CT-image-dependent anatomic center is not validated as a real anatomic center of the MM posterior root attachment. A biomechanical study using cadaveric knees will be required to determine the optimum position of the tibial tunnel in MM posterior root repair. However, the MM condition in patients with symptomatic MMPRTs may be different from that in cadaveric knees. Fourth, the aiming guide setting and tibial tunnel creation were performed by a single experienced orthopaedic surgeon (TF). The usability of these two guides was not scientifically verified by the other surgeons. Finally, there was no significant difference in the accuracy of tibial tunnel creation between these two guides.

## Conclusions

The newly developed point-contact UMR guide can enable us to create tibial tunnels for MM posterior root repairs at a favorable position as well as the MMPRT guide can. We conclude that the newly developed UMR guide system has a high accuracy in creating tibial tunnels at reliable positions during MM posterior root repairs.

## Data Availability

Data sharing was not applicable to this article as no data sets were generated or analyzed during the current study.

## Abbreviations

3D: Three-dimensional
CT: Computed tomography
ICC: Intra-class correlation coefficient
MM: Medial meniscus
MMPRT: Medial meniscus posterior root tear
PCL: Posterior cruciate ligament
UMR: Unicorn Meniscal Root
